# Gender diversity and syphilis: something's going on?

**DOI:** 10.3389/fsoc.2023.1232609

**Published:** 2023-10-17

**Authors:** Mercedes de Dios-Aguado, Aliete Cunha-Oliveira, Maylene Cotto-Andino, Pacita Geovana Gama de Sousa Aperibense, Maria Angélica de Almeida Peres, Sagrario Gómez-Cantarino

**Affiliations:** ^1^Health Center Sillería, Castilla-La Mancha Health Service (SESCAM), Toledo, Spain; ^2^Health Sciences Research Unit: Nursing (UICISA: E) and Nursing School of Coimbra (ESEnfC), Coimbra, Portugal; ^3^CEIS-20 da Universidade de Coimbra, Coimbra, Portugal; ^4^Department of Modern Languages, Faculty of Education, University of Castilla-La Mancha, Toledo, Spain; ^5^Nursing Institute of the Multidisciplinary Center, Universidade Federal do Rio de Janeiro, Rio de Janeiro, Brazil; ^6^Anna Nery School of Nursing, Federal University of Rio de Janeiro, Rio de Janeiro, Brazil; ^7^Faculty of Physiotherapy and Nursing, Toledo Campus, University of Castilla-La Mancha, Toledo, Spain

**Keywords:** homosexuality, transsexuality, syphilis, gender, sexuality, nursing history

## Abstract

**Introduction:**

This study aims to analyze the influence of syphilis among people with sexual and gender diversity, different from the binary dimension.

**Materials and methods:**

A systematic review was conducted as a method to address the objective of the study, based on the Dialectical Structural Model of Care (DSM), to obtain the phenomenon from the perspective of cultural history.

**Results:**

In this review the analysis of 129 documents, of which 22 texts were used. The construction of sex and gender in Western civilization is based on the Judeo-Christian tradition, which permitted many people throughout history to be persecuted and mistreated for living a lifestyle different from that dictated by religious and traditional canons. Therefore, throughout history, gender-diverse people, sexual minorities, and prostitutes have suffered segregation, mockery, aggression, and health problems, including syphilis.

**Conclusions:**

Despite having a treatment and cure, syphilis has stood the test of time and has remained a secret pathology that is obscure and difficult to detect disease, which is still very much present in people of all social classes. It is necessary to review history to understand the reasons why syphilis is still prevalent in different societies today.

## 1. Introduction

For centuries, the construction of sex and gender in Western civilization was based on the Judeo-Christian tradition, which devised the figure of Eve to describe female sexuality. Considering this female figure, women were held responsible for the errors of humanity (Northurp, 1998). The discourse on the female sex, inspired by the medical knowledge of Hippocrates, Galen, Avicenna, Averroes, and Juan Huarte de San Juan, was based on analyses of the anatomy and physiology of women from a modified version of men, which, for years, allowed women to be considered as imperfect beings (Vázquez Jiménez, 2015).

Between the 16th and 17th centuries, owing to the curiosity to understand female sexuality, physician-anatomists Mateo Realdo Colombo, Gabriele Falloppio, and Caspar Bartholin investigated the anatomy and physiology of the female genital organs through dissection. Through their treatises on anatomy, the immeasurable division of the two sexes began to be proclaimed with scientific rigor (Lafortune and Berriot-Salvadore, 1994).

By the 18th century, women were no longer considered an inferior version of men, but just different beings. The binary dimension of the male-female gender was consolidated. Doctors showed an interest in female pathologies, although they considered that illness in women stemmed from a rupture with nature since the healthy and happy woman was the one who complied with the established social order and maintained sexual relations within marriage (Vázquez Jiménez, 2015; Pérez, 2017). Against this background, it is worth highlighting the importance generated in the process of how a person identifies themselves; they incorporate objects that serve as referential models during the process of forming their identity. In fact, the cultural forms of life remain organized by the heterosexual matrix. Consequently, heterosexuality is installed as a legitimate mode of erotic choice. Therefore, heterosexuality is formed through norms that, to some extent, derive from homosexual relations, forcing their suppression (Butler, 2003).

For centuries, sexual morality conformed to biblical laws, which were summarized by Philo of Alexandria, a Jewish-Hellenist philosopher who interpreted the biblical ten commandments in his treatise De specialibus legibus. The philosopher made a profound and important reflection on the forms of interpersonal relationships and sexual morality. In the text, sexual relationship is explained through metaphorical words or with a euphemistic sense, and his theses come from the deep reflection he made on the forms of interpersonal relationships and sexual morality, a fact that allows us to know the sexual behavior of the time and the different existing gender roles (Pérez, 2017).

It is essential to understand that the term “sex” refers to the inherent biological differences between individuals as men and women, closely related to their primary and secondary sexual characteristics. However, the concept of “gender” encompasses the cultural construct intertwined with aesthetic attributes, values, and perceptions related to what is considered feminine or masculine. In this context, gender stereotypes involve patterns, roles, and personality traits that have historically been associated with the representation of being female or male within a specific society (Atienza Macías and Armaza Armaza, 2014). It was not until the 18th century that women were recognized as beings with their own identities, so it is worth asking what happened in relation to sexual and gender diversity other than the binary dimension. It is even possible to wonder if, until that century, medical science did not become interested in female pathologies in relation to sex-related diseases. There could have been a lack of interest or intolerance to research, especially when sexual relations were maintained within marriage in accordance with the sexual morality governed by the Judeo-Christian tradition. Furthermore, sex and desire are mechanisms of power that, by transmitting sexuality, produce repressive or oppressive systems where one exerts greater dominance over the other (Foucault, 2008). These roles and stereotypes play a crucial role in society, as they have historically established specific boundaries and functions for men and women. However, the problem lies in the fact that these patterns continue to perpetuate lifestyles that originated in the early stages of society without considering the sociocultural evolutions that have taken place over the centuries (Swaab et al., 2021). According to data from the Spanish Ministry of Interior, in 2019, there were 278 hate crimes related to sexual orientation or gender identity, with an increase of 8.6% compared to 2018 (News Mundo, 2021).

Syphilis, a sexually transmitted infectious disease whose causal agent is the bacterium *Treponema pallidum*, has a torpid evolution and is being transmitted by direct contagion or via the placenta. It has several stages, the first of which is characterized by the presence of a non-painful open ulcer called a chancre, the location of which depends on the different sexual practices and may be present on the genitals, mouth, skin, rectum, or vagina (Janier and Caumes, 2012; Pitche, 2022). If treatment is effective, the lesion heals in ~4–6 weeks. If the medication fails to achieve its purpose, between 2 and 8 weeks after the chancre has healed, syphilis progresses to a secondary period, where a cutaneous-mucosal rash appears with non-painful lesions affecting the skin, mucous membranes, palms of the hands, and soles of the feet. At this stage, *Treponema pallidum* pass into the bloodstream (Capdevila and Fernández, 2007; Pitche, 2022). Therefore, if treatment is inadequate, there is systemic and visceral involvement, causing fever, malaise, arthralgia or myalgia, swollen lymph nodes, headache, weight loss, and fatigue. It can even result in jaundice, splenomegaly, glomerulonephritis, and bone involvement. When treatment remains inadequate, the person continues to have syphilis, even if no symptoms are present; this phase is called latent syphilis and can last for years (Palacios Muñoz et al., 2006; Capdevila and Fernández, 2007), and in this stage, *T. pallidum* colonizes and reproduces, resulting in a circular ulcer called a gum. At this stage, there may be involvement of bones, skin, mucous membranes, nervous tissue, brain and meninges, heart, and arteries (Ministerio de Sanidad and Dirección General de salud Pública, 2021). The infection may be latent for 10–30 years, giving rise to late syphilis, a stage characterized by tabes dorsalis and neurosyphilis, a disease that evolves into syndromes such as psychosis, mania, dementia, and even epilepsy (Capdevila and Fernández, 2007; Janier and Caumes, 2012; Pitche, 2022).

This article aims to analyze the influence of syphilis among people with sexual and gender diversity, other than the binary dimension from the history of nursing. The question guiding the study is: How were relationships between people with sexual and gender diversities, other than the binary dimension, and syphilis established? The justification for the study is based on knowing what has happened and is happening within this group of people in relation to syphilis, especially when its prevalence worldwide is very high in all social groups.

## 2. Materials and methods

### 2.1. Study design

In this study, a systematic review was undertaken to address the objective of the study. These reviews are an ideal tool for determining the scope of a set of publications on a given topic. They indicate the volume of publications and studies available. In addition to acquiring a global vision of the documents, the reviews allow researchers to evaluate, synthesize, and critique the evidence inherent to the objective of the study (McFarlane, 1988; Munn et al., 2018). The Dialectical Structural Model of Care (DSMC) (Siles González, 2005) has been used because of its relevance in delving into the cultural and social roots of structures that are particularly linked to sexual and gender diversity, as opposed to the binary dimension. This model identifies the functional dynamics of structures, allowing the analysis of the causes that lead to their changes.

**Figure 1:**
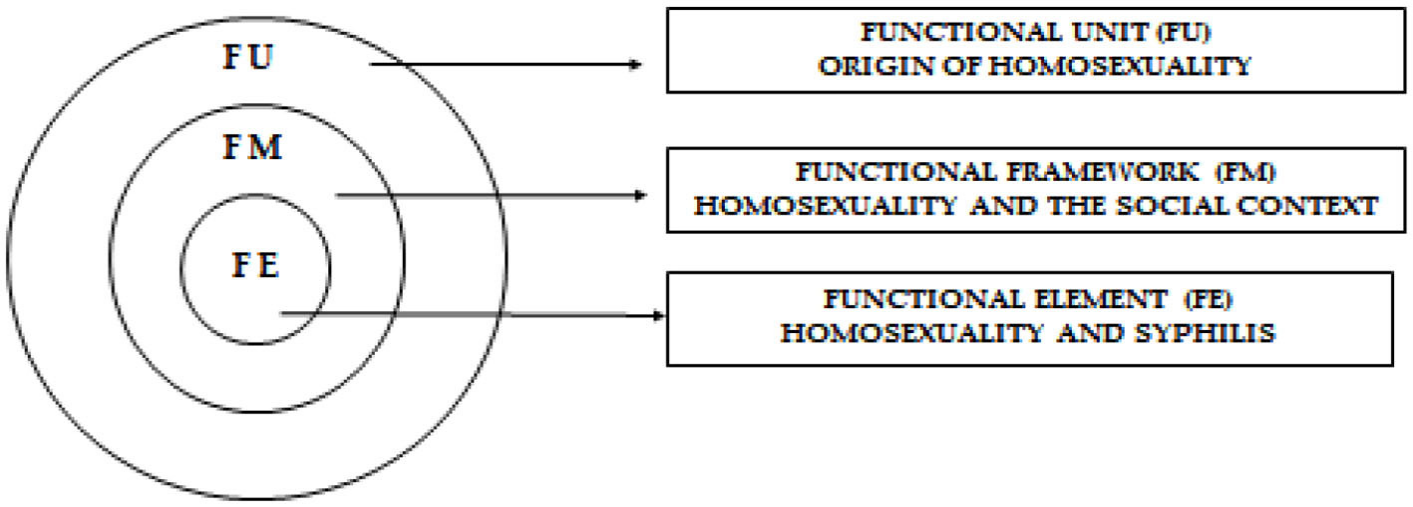
MEDC methodology developed by the authors.

The Multiple Event Detection and Classification (MEDC) methodology is based on structures that serve as support for the process of ordering and analyzing the data. In this research, its application is important due to the social, cultural, and care study in which we are involved. Therefore, the structures applied to it are the following: (1) functional unit (F.U), where the origin of sexual and gender minorities in the various social systems that determine sexual and gender diversity, other than the binary dimension, is addressed; (2) functional framework (F.M), related to sexual and gender minorities within the social context; and (3) functional element (F.E), which in this case reflects the relationship between sexual and gender minorities and syphilis. These structures utilize a suitable tool for the organization and analysis of the data since the aim is to obtain a vision of the historical phenomena from the perspective of cultural history (Siles González, 2004). Within this study, three thematic blocks were developed centered on the MEDC; each one of them encompassed the structures that make up this historical and cultural model (Siles González and Solano Ruiz, 2016) (Figure 1).

### 2.2. Search strategy and review process

The process of a systematic review started with an exploratory research question (Peters et al., 2015) aimed at synthesizing and systematically critiquing existing knowledge (Colquhoun et al., 2014). This review involved several steps (Levac et al., 2010).

Initially, the topic was identified, and a research question was established, which was posed within the cultural history and the culture of caregivers using the Dialectical Structural Model of Care (DSM): How were relationships between people with sexual and gender diversities, and syphilis established? The following inclusion criteria were considered: (1) publications in Spanish, English, Portuguese, and French; (2) no time restriction for publication (as this was historical research); (3) subject matter related to sexual and gender diversities; and (4) thematic selection based on the importance and relevance of the topic, as well as the development and influence of homosexuality throughout history. The exclusion criteria included the following: (1) repeated publications; (2) abstracts, editorials, chapters and books, theses, dissertations, and course completion papers; and (3) studies not related to the proposed topic.

**Figure 2:**
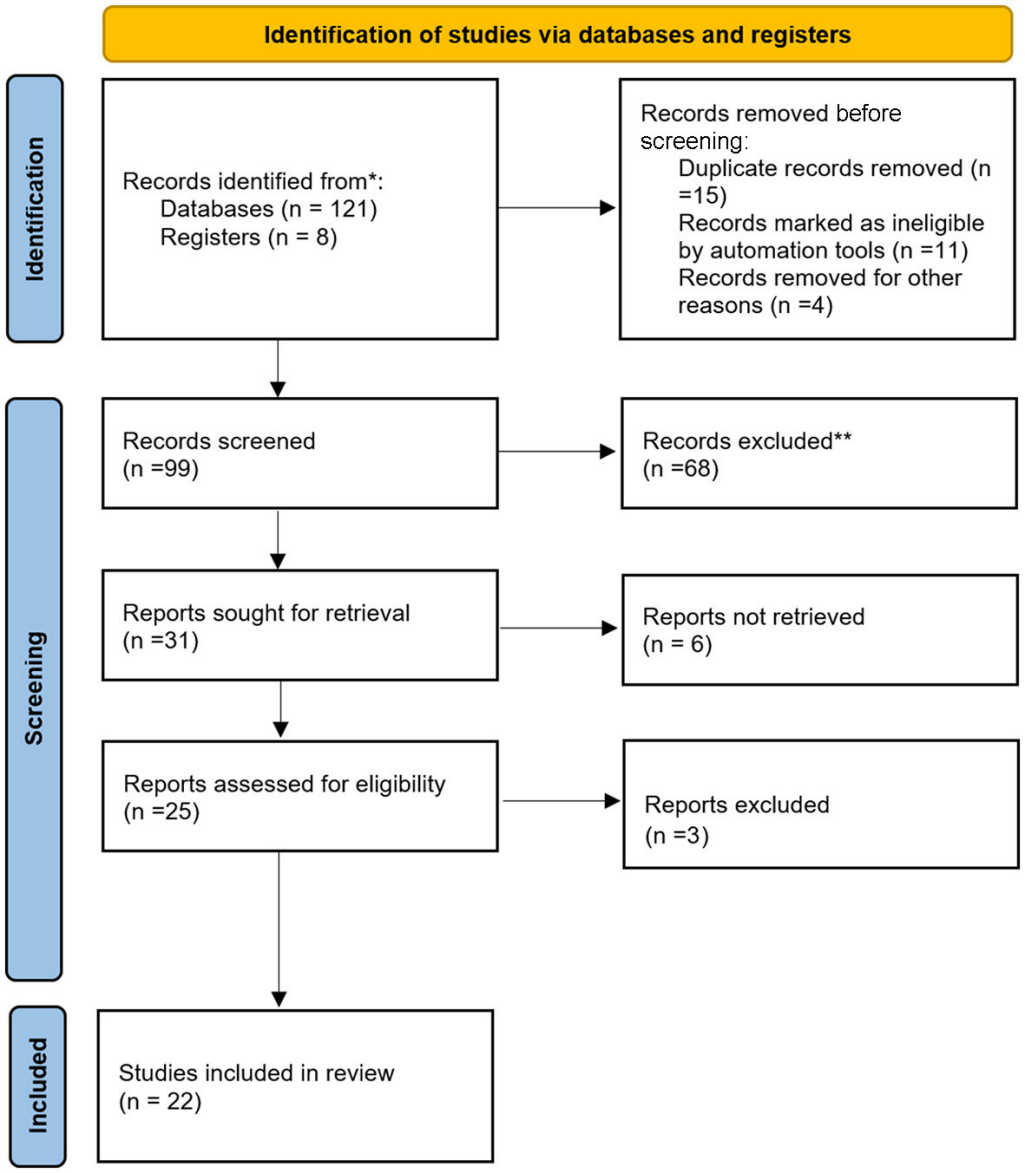
Identification of the studies, reviewed, included and used in the research. *Total studies found. **Records excluded after being examined.

The search for documentation was carried out between January and July 2022 by physically visiting the Library of the University of Castilla-La Mancha, the National Library of Spain, the Library of Castilla-La Mancha (Alcázar de Toledo), the National Historical Archive, the CSIC Library, and the World Digital Library (UNESCO). Several databases were consulted, including the following: (1) Latin American Health Bibliographic Database (CUIDEN); (2) PubMed; (3) Scopus; (4) Science Direct; and (5) Google Scholar. MeSH and DeSH terms were used to carry out a more exhaustive and advanced search using the Boolean operators “[AND]” and “[OR]”. Additionally, even word-word combinations were used where appropriate to reflect the syntax and search rules common to individual databases. The descriptors were as follows: sexual and gender minorities, transgender persons, gender diversity, syphilis, sexuality, and history of nursing. Due to the diversity of data collected from newspapers, books, digitized documents, and manuscripts, the research corpus was composed of a set of documents containing the core phrase. For the analysis of this corpus of documents, a flow chart was constructed following the PRISMA strategy, which allowed a synthesis of the information provided by each document and, consequently, the analysis of the data (Figure 2).

### 2.3. Data analysis

The documentary analysis was carried out systematically following the objective of the study. The steps followed in the analysis were as follows: (1) thematic linking; (2) preliminary classification of the documents based on inclusion and exclusion criteria; (3) selection of relevant information; and (4) interpretation and comparison of the results. The selected material was analyzed from the point of view of the three thematic blocks of study, each of them encompassed in the DSMC structures. These included the following: (1) the origin of sexual and gender minorities; (2) sexual and gender minorities within the social context; and (3) sexual and gender minorities and syphilis. The first, second, and third authors carried out general data collection. The fourth author examined the findings in depth, and the fifth and sixth authors identified the thematic blocks under the DSMC structures of functional unit, functional framework, and functional element. Discrepancies were resolved by consensus among the researchers.

## 3. Results

As can be observed in the PRISMA strategy, 129 publications were identified in the database. However, once the documents were filtered, 99 studies were selected, of which 31 were adjusted to the search strategy, although, to undertake this study, only 22 were useful. It should be noted that most of the documents were published in the 21st century, probably due to the current interest in understanding people with sexual and gender diversities beyond the binary dimension. The findings from the 22 selected studies were categorized into three common thematic blocks for further analysis to provide clarity and consistency in the topic of study. The main thematic lines focused on three common thematic lines: (1) understanding the views and perspectives of the origin of sexual and gender minorities, which was the focus of 10 studies; (2) exploring sexual and gender minorities in the social context, which was the focus in five studies; and (3) determining sexual and gender minorities and syphilis, which was discussed in seven studies.

### 3.1. Origin of sexual and gender minorities

Currently, there is no agreement on the origin of homosexuality. Some researchers believe that, in primitive civilizations, it was accepted and permitted as part of the idiosyncrasies of human beings in those civilizations (essentialist current), while other researchers consider homosexuality to be a social construct introduced in the Middle Ages because of the existing dichotomy between homo-social and hetero-social relationships (constructionist current) (Solana, 2018).

Current essentialist scholars argue that, in Egypt, Greece, and Rome, interpersonal relationships were allowed as long as they satisfied the pre-established moral canons and adapted to the rational ethical principles and social convictions of the time, even if they only appeared to be in conformity and the true motive of the relationship was hidden (Pulecio Pulgarin, 2009). Plato, in his work, *The Banquet*, states that it is possible to understand that there are men who sexually seek other men, women who prefer sexual contact with women, and men who desire women and women who desire men (Solana, 2018).

However, current constructionist scholars argue that the concept of homosexuality emerged in the West during the 12th and 13th centuries. According to them, closed communities of men existed where intimate coexistence between members was the backbone of a homo-social relationship. Such relationships fostered virile friendships, whose masculine ideal was the warrior, a man who lived apart from women (Soto Buenaventura, 2021).

During these centuries, feudal society promoted the male-female love relationship, and sexual practices were oriented toward the lady's gallantry, even though the woman was considered a commodity, the property of the masters, the father, and the husband. With gallantry came the concept of courtly love, the basis of the troubadours' lyrics, which exalted the permanent suffering of the lovers in their impossible love and passion and where the love relationship went beyond good and evil. Consequently, the dichotomy of homosexual relationships and heterosexual relationships emerged during the 12th and 13th centuries (Di Gerónimo, 2012).

The Christian Church, between the Second Lateran Council of 1139 and the Fourth Lateran Council of 1212, attempted to weaken the existing exaltation of love within the closed communities of the religious orders. This led to the persecution of marriages and concubinages of priests, deacons, sub-deacons, monks, and nuns. Adulthood was viewed as a sin since male-female love was only allowed within the conjugal framework. Further, with the intention of limiting homo-social relationships within religious orders, he introduced the accusation of sodomy to condemn any loving relationship that was not male-female. Incidentally, the term sodomy comes from the story of Sodom and Gomorrah in the Old Testament (Soto Buenaventura, 2021).

During the 13th and 14th centuries, the Catholic Church, through the tribunal of the Holy Inquisition, tortured people in same-sex relationships accused of sodomy and condemned them to death at the stake for being guilty of a nefarious sin. Such persecution was widespread until the 18th century (Boswell, 1998; Torquemada Sánchez, 2014).

In 17th and 18th century England and France, people in same-sex relationships gathered in so-called fun houses— places where social and sexual norms were challenged—as pleasure was at its peak and there was unbridled passion (Cancino Barffusón, 2012). However, to get around the accusation of sodomy, euphemistic expressions such as libertine, romantic friendship, Malthusian partner, and sexual invert were used. Sexual attraction was considered an autonomous biological and psychic instinct (Foucault, 2008; Cancino Barffusón, 2012). In his play, *The Bostonians*, Henry James recreated an emotional union between two independent women, which gave rise to the term “Boston marriage” (Alventosa del Río, 2008; Beteta Martín, 2012). During this time, only the term “homo-social practices” existed, for it was not until 1869 that the term homosexual was coined to refer to a specific type of person (Cancino Barffusón, 2012).

In the 19th century, people in same-sex relationships began to be targeted for their gestures, their manners, their way of dressing, and their anatomy, morphology, and facial expressions (Foucault, 2008). With the advent of sexology, homosexuality was considered a pathological disorder. As a result, the person who felt affective and sexual attraction for a person of the same sex and behaved in an unconventional way was treated with therapeutic techniques of little validity. With the rise of totalitarian regimes in the 20th century, homosexuality came to be persecuted and strongly repressed, and people in same-sex relationships were considered a danger to society. In this context, lesbianism was considered a disease among female prostitutes (García Valdés, 1980; Alventosa del Río, 2008). With the first surgical interventions on transsexuality, a sub-specialty emerged within sexology whose purpose was to attend to the needs of transsexuality (Beteta Martín, 2012) (Table 1).

**Table 1:** Sexual and gender minority origin.

**Authors**	**Year**	**Document**	**Topic**
Solana, M.	2018 Artícle	El debate sobre los orígenes de la homosexualidad masculina. Una revisión de la distinción entre esencialismo y construccionismo en historia de la sexualidad.	It is understandable that men desire women and women desire men (Plato).
Pulecio Pulgarin, J. M.	2009 Artícle	Filosofía y diversidad sexual: aportes para una lectura de la constitución colombiana en clave de género.	Essentialist tendency: Egypt, Greece and Rome, in interpersonal relationships satisfactory to the canons, adapted to rational ethical principles and consented to them.
Soto Buenaventura, J. D.	2021 Artícle	La invención de la cultura heterosexual: Louis-Georges Tin.	Essentialist tendency: Egypt, Greece and Rome, in interpersonal relationships satisfactory to the canons, adapted to rational ethical principles and consented to them.
Di Gerónimo, M.	2012 Article	El amor cortés: Escenas amorosas que sostienen mundos. Caso Borges.	Feudal society encouraged male-female love relationship (XII-XIII) the dichotomy of homosexual relationship or heterosexual relationship emerged.
Boswell, J.	1998 Book	Cristianismo, Tolerancia Social y Homosexualidad, Los gays en Europa occidental desde el comienzo de la Era Cristiana hasta el siglo XIV.	Catholic Church (XIII-XIV) through the tribunal of the Holy Inquisition tortured homosexual persons.
Torquemada Sánchez, M. J.	2014 Artícle	Homosexualidad femenina y masculina en relación con el delito de sortilegios.	Persecution by the Church (Holy Inquisition) was very present until the 18th century.
Cancino Barffusón, S. R.	2012 Book	Permanencias, cambios y tensiones en el proceso de visibilización de la homosexualidad masculina en Xalapa, Veracruz.	The term homosexual was coined (1869). Meeting place, houses of entertainment, where social and sexual norms were challenged.
Beteta Martín, Y.	2012 Artícle	De la tradición sáfica a los círculos triádicos: la búsqueda de las identidades lésbicas desde una perspectiva histórica (De la antigüedad clásica a la edad moderna).	Bostonian marriage: emotional union between two independent women.
Alventosa del Rio, J.	2008 Law	Discriminación por orientación sexual e identidad de género en el derecho español.	Late 19th century lesbianism considered a disease of prostitute women.
García Valdés, A.	1980 Book	Historia y presente de la homosexualidad: análisis crítico de un fenómeno conflictivo.	With the rise of totalitarian regimes (20th century) homosexuality was persecuted and strongly repressed, people in same-sex relationships being considered a danger to society.

Following the spontaneous emblematic moment of homosexuality that occurred in the early hours of 28 June 1969 in the Stonewall Inn (New York), stigmatization, discrimination, and homophobia have been combated. The Stonewall riots that followed are often cited as the first instance when the gay community fought against a system that persecuted non-normative people (Cancino Barffusón, 2012).

### 3.2. Sexual and gender minorities within the social context

The repression of certain types of sexual behavior in both private and public life began in feudal Europe. A redefinition and updating of social values followed, which led to the persecution of behavior that violated pre-established norms. Those who challenged the values and/or deviated from pre-established norms were called heretics and known as sodomites in the 13th century. They were the target of intolerance and persecution, resulting in the phenomena of interpersonal violence, such that anyone whose behavior deviated from Christian morality was socially marginalized during the 14th and 15th centuries (Bazán Díaz, 2007).

In an intolerant society, as described above, people who were rejected or persecuted for their sexual behavior for centuries lost their rights to develop their human potential and were forced to repress their feelings for fear of being denounced or scrutinized by people whose recognition or social position gave them power (Porras Gallo, 2002).

The demographic growth of the 19th century, a consequence of the Industrial Revolution, produced suburbs on the outskirts of cities inhabited by a diverse group of people, including those marginalized by their sexual behavior. This gave rise to slums or Chinatowns—population nuclei where people lived in overcrowded conditions—with precarious economic means and with the need to seek daily sustenance. The only recourse for men and women in these places who intended to improve their economic condition was practicing prostitution (Porras Gallo, 2002; Huard, 2016).

In Spain, during the Franco dictatorship, prostitution (both male and female) was the economic engine of these neighborhoods despite police controls. The homophobic discourse was developed during this period with the approval of the Church and state institutions. Anyone whose gender identity did not fit the heterosexual model was forced to hide and repress their feelings to the point of becoming invisible for fear of being arrested for public scandal, as homosexuality was criminalized as per the 1954 Law on Vagrants and Thugs. This law was enacted with the aim of curbing and controlling prostitution (Huard, 2016; Díaz, 2019).

Consequently, venereal disease among the homosexual population was kept a secret, especially in Chinatown, which provided discretion and a certain level of tolerance toward permissive behavior, making it easier for the homosexual community to take refuge there. However, affluent members of the homosexual population met in more sanitized and hygienic environments. As a result, the Drugstore in Barcelona's Gracia district and Madrid's Chueca district emerged as popular gathering places for the homosexual community. Today, these areas are acknowledged for their association with homosexuality, where it still remains an integral part of the respective neighborhoods (Herrero-Brasas, 2001; Díaz, 2019) (Table 2).

**Table 2:** Social context: sexual and gender minorities.

**Authors**	**Year**	**Document**	**Topic**
Bazan Díaz, I.	2007 Artícle	La construcción del discurso homofóbico en la Europa cristiana medieval. En la España medieval.	People whose behavior was far from Christian morality were socially marginalized during the 14th and 15th centuries.
Porras Gallo, M. I.	2002 Artícle	Un acercamiento a la situación higiénico-sanitaria de los distritos de Madrid en el tránsito del siglo XIX al XX.	Industrial Revolution, produced suburbs outside cities: inhabited by people marginalized by their sexual behavior where people lived in overcrowded conditions (slums).
Huard, G.	2016 Artícle	Los homosexuales en Barcelona bajo el franquismo. Prostitución, clase social y visibilidad entre 1956 y 1980.	Under the Franco dictatorship (1939-1975), prostitution, both male and female, was the economic engine of marginalized neighborhoods, with the approval of the Church and State institutions.
Díaz, A.	2019 Article	Los “invertidos”: homosexualidad (es) y género en el primer franquismo. Cuadernos de historia contemporánea.	Law on vagrants and malefactors (1954). Law enacted with the aim of curbing and controlling prostitution, including people in same-sex relationships.
Herrero-Brasas, J. A.	2001 Book	La sociedad gay: una invisible minoría.	People in same-sex relationships from the wealthy classes met in places with greater guarantees of hygiene. In places where homosexuality was and is part of the idiosyncrasy of the neighborhood.

The Law on Vagrants and Thugs was selectively applied with class criteria since individuals in homosexual relationships belonging to powerful families were excluded from it. Therefore, a very high number of those judged by the courts of the time for homosexuality belonged to the most disadvantaged social groups; it was exceptional for someone belonging to the upper social class to be sentenced to prison for homosexuality (Herrero-Brasas, 2001; Huard, 2016; Díaz, 2019). The Law on Vagrants and Thugs was repealed on 4 August 1970 and replaced by the Law on Dangerousness and Social Rehabilitation, which remained in force until 26 December 1978. However, the homosexual community was no longer convicted under this law, though they could be arrested under the Public Scandal Act. People with sexual and gender diversity, different from the binary dimension, did not stop being persecuted until 26 December 1978, when the first president of democracy, Adolfo Suárez, signed a modification of the Law of Dangerousness and Social Rehabilitation, which eliminated homosexuality from its text, thus decriminalizing it in Spain (Herrero-Brasas, 2001).

### 3.3. Sexual and gender minorities and syphilis

In his writings, Hippocrates describes a disease associated with vagrancy, overcrowding, immorality, and a lack of hygiene, which is similar to the symptoms of syphilis. However, some researchers believe that syphilis arrived in Europe after Columbus' third voyage in 1493; on arriving in Spain, Columbus reported that one of his crew members had contracted an infection on the island of Hispaniola (Frith, 2012). The first known treatment for syphilis consisted of the following: applying guaiac resin, meticulous removal of the syphilitic patient's secretions from their lesions, and applying mercury preparations by the active rubbing of the patient's skin with a Neapolitan ointment or gray ointment (Leitner et al., 2007). The discovery of the causative agent of syphilis in 1905 by Fritz Schaudin and Erich Hoffman marked the beginning of effective treatment for this disease. August von Wassermann's understanding of the humoral reaction for the serological diagnosis of syphilis in 1906 furthered progress in this field. Alexander Fleming's discovery of the *Penicillium notatum* bacterium in 1928 also contributed to the advancement of treatment for syphilis. These three milestones changed the course of the disease (Ros-Vivancos et al., 2018).

Syphilis was the cause of death of great music composers, scientists, and politicians who were associated with prostitution and homosexuality. Discretion over this influenced the spread of the most non-visible stages of the disease. Therefore, in the 19th century, with the understanding of the importance given to hygiene, attempts were made to regulate prostitution, even advocating its abolition, while any manifestation of homosexuality was strongly repressed. During the first half of the 20th century, there was strict sanitary control of brothels. Meanwhile, medical treatises of the time supported sex education following Judeo-Christian morality with the intention of eradicating syphilis and, at the same time, promoting sexual relations within the conjugal framework. However, even with this tight control, syphilis was not eliminated among the heterosexual or homosexual population (Ros-Vivancos et al., 2018).

The discovery of penicillin and the control of prostitution considerably reduced the prevalence of syphilis and the incidence of the more serious forms of the disease, such as cardiovascular syphilis, neurosyphilis, and congenital syphilis. However, the disease continues to be called the great simulator because every 10 years or so, there are upturns in incidence. Therefore, with the sexual revolution, a widespread use of contraceptives, the liberalization of homosexual relations, and injecting drug use, syphilis continues to be a worldwide problem among the heterosexual and homosexual populations (Capdevila and Fernández, 2007) (Table 3).

**Table 3:** Syphilis: sexual and gender minorities.

**Authors**	**Year**	**Document**	**Topic**
Frith	2012 Artícle	Syphilis its early history and treatment until penicillin and the debate on its origins. J Mil Veterans Health.	Syphilis arrived in Europe after Columbus' third voyage in 1493.
Leitner R. M. C., Körte, C., Edo, D., Braga, M. E.	2007 Artícle	Historia del tratamiento de la Sífilis.	First treatments of syphilis: guaiac resin, mercurial preparations, Neapolitan ointment or gray ointment.
Ros-Vivancos C., González-Hernández, M., Navarro-Gracia, J., Sánchez-Payá, J., González-Torga, A., and Portilla-Sogor, J.	2018 Artícle	Evolución del tratamiento de la sífilis a lo largo de la historia.	Serological diagnosis of syphilis: discovery of the *Penicilium notatum* bacterium (1928) by Alexander Fleming.
Capdevilla E. F., Fernández, M. M.	2007 Artícle	Evolución del tratamiento de la sífilis a lo largo de la historia.	Stages and course of the disease.
Palacios Muñoz, R De la Fuente Aguado, J., Murillas Angoiti, J., Nogueira Coito, J. M. Santos González, J.	2006 Artícle	Sífilis e infección por el virus de la inmunodeficiencia humana.	Latency of disease (10–30 years): Late syphilis (tabes dorsalis and neurosyphilis).
Repiso, B., Frieyro, M., Rivas-Ruiz, F., De Troya, M	2010	Uso preservativo y número de parejas sexuales en hombres que tienen sexo con hombres con sífilis.	Prevention and control of syphilis: reduction of incidence, early diagnosis to prevent associated comorbidities.
Ministerio de Sanidad C y BS, FELGTB.	2021	Recomendaciones para la atención de personas trans en el ámbito sanitario	Care and recommendations in the treatment of transgender people.

The general objectives of syphilis prevention and control are to reduce the incidence of this infection, to promote early diagnosis to prevent associated comorbidities, and to reduce discrimination against people suffering from this pathology (Palacios Muñoz et al., 2006; Repiso et al., 2009). In Spain, it is difficult to encourage a person for diagnosis among the heterosexual population, but it is almost impossible among the homosexual population, especially when syphilis is perceived as a social stigma, and the infected person does not present symptoms that would make it necessary to carry out a diagnostic test. These two factors, together with the difficulty of accessing resources, the lack of information, and the inconvenience of contact tracing, mean that the chain of transmission is not stopped, and syphilis goes undetected in the heterosexual and homosexual populations (Ministry of Health, 2021).

## 4. Discussion

The construction of sex and gender in Western civilization is based on the Judeo-Christian tradition, which devised the figures of Adam and Eve to describe sexuality and allowed many people throughout history to be persecuted, abused, and burned at the stake simply for living as their mind, but not their body, dictated.

Against this background, it is worth mentioning that the life of Elena/o de Céspedes (1545–1588), a male transsexual who, as a woman, was mentally convinced that she belonged to the opposite sex and was trapped in a female body. With the help of surgery, she managed to modify her anatomy to adapt it to the gender she really wanted. Once she accomplished the feat (sexually male), Céspedes married María del Caño, and for more than a year, they lived together in the town of Yepes (Toledo, Spain) without their sex life being a cause for suspicion (Barbazza, 1984).

However, as a result of some quarrel, the couple were denounced to the court of the Holy Inquisition, and on 18 December 1588, the Auto Public de Fe was held in the Plaza de Zocodover in Toledo, where Céspedes abjured herself and, by way of mockery, went through the streets of Toledo wearing insignia that showed her gender diversity. Her sentence was exemplary, and she was confined for 10 years in a hospital. During this time, Céspedes worked without pay in the hospital infirmary, which earned her freedom from the stake, but in the end, they did not achieve her goal of being recognized as a man (Barbazza, 1984).

Throughout human history, syphilis has been closely linked to sexual promiscuity, and for centuries, it was considered a shameful disease, and people who suffered from it were socially rejected. Therefore, people with gender diversity, sexual minorities, and prostitutes suffered segregation, mockery, and aggression. However, medically, they lacked understanding, as syphilis was silenced among this group so as not to suffer discrimination. As a result, when they did seek help to treat their disease, the disease was so advanced that it caused their death, a fact that was very common in the 19th century (Ros-Vivancos et al., 2018). In the First World War, the spike in the disease caused thousands of soldiers to contract syphilis. Given that syphilis was considered a shame, the French army created and promoted the use of “washing stations” where the soldiers would wash their hands after having sexual relations. Therefore, when a soldier was questioned in the event of any symptom of disease if he had not followed this process, the medical team would start treatment for syphilis (Volcy, 2014).

Since the end of the 19th century, lesbianism has been considered a disease among female prostitutes (Alventosa del Río, 2008). Therefore, at the beginning of the 20th century, when the abolitionist campaign (that started in England during the second half of the 19th century) began in Spanish society, it culminated in a new policy that promoted strict control of prostitution. In the era of the French Second Republic, the decree of 28 June 1935 was passed, abolishing prostitution, considering it an illicit livelihood (Rivas Arjona, 2013).

Although the objectives of the 1935 decree included the repression of sexual and gender minorities, as well as the control of syphilis, during the Franco dictatorship, prostitution (male and female) was the economic engine of certain neighborhoods and created a spike in syphilis. It increased in prevalence until the arrival of acquired immunodeficiency syndrome (AIDS) at the end of the 20th century (Huard, 2016; Díaz, 2019).

**Figure 3:**
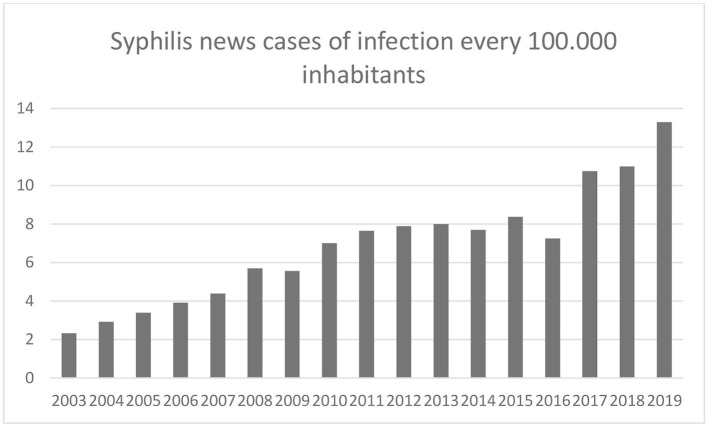
Evolution of syphilis cases (2003–19).

Many centuries have passed since Céspedes and several years since the First World War, but little has changed in society. Since 2003, there has been a clear increase in syphilis cases in Spain, from 2.32 cases every 100,000 inhabitants to 13.29 cases in 2019, including data showing a male bias, with 89% of infections detected in male populations in the 20–24 age group (Ministry of Health, 2021) (Figure 3).

Therefore, something is going on when many Lesbians, Gay, Transgender, Transsexual, Transgender, Bisexual, Queer, and Intersex (LGBTQIA+) people cannot continue to live according to their minds or their bodies. Some of them are shouted at as “faggot” while being killed, as happened to Samuel Luiz, a nurse in La Coruña. He was killed simply for being near a discotheque, enjoying his time off with his friends. Luiz was assassinated in a free, democratic, and confessional country. Many centuries separate the lives of Céspedes and Luiz, but neither of them achieved their purpose, which is to live according to their minds, not their bodies. Although, indeed, healthcare for the transgender population in Spain has undergone significant advances in recent years, owing to the approval of regional and national laws that guarantee the rights of LGTBI people, these laws have allowed these people access to more inclusive health services that respect their gender identity (Navarro-Pérez et al., 2015).

Regardless, even today, discrimination is perceived by transgender patients; 48.8% of transgender patients have had delayed or canceled medical appointments. Of these patients, 16.7% said that they are afraid of not being treated with respect by health professionals when seeking care. There is a requirement for an environment that highlights the need for quality care without stigmatization and adequate and updated training for health professionals, including an understanding of diverse gender identities and expressions (Mulió Álvarez and Europe, 2019; Crespo Ibor and Almudéver Campo, 2020).

This study is limited by its historical focus since, for centuries, syphilis was one of the most feared social ills. While the disease has been considered a minor problem in the West for decades, it has re-emerged strongly in Europe and the United States. As a result, new evaluations of its functioning and health care are emerging, making it difficult to generalize its results. However, this study promotes a contribution that aims to encourage nursing teachers to reflect on educating themselves on diverse sexualities in order to create strategies to transform identifiable barriers into opportunities to improve the quality of teaching on this topic.

## 5. Conclusions

Over the years, gender identity has been socially interpreted in many ways, with the most prominent being behaviors related to male and female homosexuality. The influence of the Church in the moral determination of Western society established that sexual relations could only be maintained within marriage. The sexual couple was constituted within the male-female binomial, with the male and female roles being perfectly defined along with their sexual behavior. There was no room for a homosexual relationship in this construct. Therefore, if such a relationship existed, it was considered a result of some mental disorder of the man or the woman. Under this interpretation, for centuries, people with sexual and gender diversity suffered persecution, rejection, and punishment, either as heresy or criminality.

This article shows the effect of society on the lives and behavior of people who do not conform their gender identity to their birth sex. Throughout history, people with sexual and gender diversity, other than the binary dimension, have suffered segregation, ridicule, assault, and health problems, including syphilis. Despite treatment and cure, this pathology has remained hidden and difficult to detect and is still very pervasive among people from all walks of life. The repression of prostitution and other behaviors considered sexually deviant failed to eradicate syphilis. Therefore, it is necessary to review history to understand why no progress was made in this field of health in relation to other periods when it was assumed that only debauched and marginalized people suffered from syphilis.

This study reveals the existence of intolerance or acceptance by the public toward the LGBTQIA+ community, a situation that sometimes generates the difficulty of a specific approach to the health of this group despite the existence of specific mechanisms for health care. As a result, syphilis continues unabated and manages to increase its incidence every 10 years.

Therefore, this study should serve as an indicator and reminder for LGBTQIA+ people to have a greater number of initiatives where specific health programs are promoted to prevent syphilis from remaining a secret disease among this group.

## Data availability statement

The original contributions presented in the study are included in the article/supplementary material, further inquiries can be directed to the corresponding author.

## Author contributions

MD-A, SG-C, and AC-O: conceptualization. AC-O, PG, and MA: methodology. MD-A and MA: validation. MD-A, SG-C, AC-O, and PG: formal analysis. MD-A, SG-C, AC-O, and MA: investigation. MD-A and SG-C: writing—original draft. MD-A, AC-O, PG, and MA: writing—review and editing. SG-C and AC-O: supervision. PG: project administration. All authors have read and agreed to the published version of the manuscript.
